# Hydration and Microstructure of Steel Slag as Cementitious Material and Fine Aggregate in Mortar

**DOI:** 10.3390/molecules25194456

**Published:** 2020-09-28

**Authors:** Wu Jing, Jinping Jiang, Sha Ding, Ping Duan

**Affiliations:** 1 School of Civil Engineering and Architecture, Wuhan University of Technology, Wuhan 430070, China; jing_wu2020@126.com; 2 Wuhan Hanyang Municipal Construction Group Co., Ltd., Wuhan 430050, China; jol007@163.com; 3 Key Laboratory of Geological Survey and Evaluation of Ministry of Education, Faculty of Materials Science and Chemistry, China University of Geosciences, Wuhan 430074, China; jinping_jiang@163.com; 4 Guangxi Key Laboratory of New Energy and Building Energy Saving, Guilin University of Technology, Guangxi 541004, China; 5 Zhejiang Institute, China University of Geosciences (Wuhan), Hangzhou 311305, China

**Keywords:** steel slag, fine aggregate, cementitious material, hydration

## Abstract

Due to the low hydration activity and poor volume stability, extensive steel slag utilization is restricted. In this paper, the hydration process and microstructure of alkali-activated materials with steel slag as a cementitious material and fine aggregate were studied. The phase composition and micro-morphology of hydration products were measured using XRD, NMR and SEM. The response relationship between microstructure and mechanical properties during hydration was revealed. The results show that the main hydration products of the alkali-activated steel slag powder-granulated blast furnace slag powder cementitious system are Ca(OH)_2_ and calcium aluminosilicate hydrate (C-A-S-H) gel. With the progress of hydration, the amount of calcium silicate hydrate (C-S-H) gel and the average molecular chain length increase, Al[4]/Si decreases, while C/S increases first and then decreases, and the structure of cement paste becomes much more compact. The interface between steel slag sand and cement paste is denser than that of river sand, since the hydration occurs on the surface of steel slag sand, which leads to the formation of C-A-S-H gel and Ca(OH)_2_. As a result, the compressive strength of concrete prepared by steel slag sand is higher than that of river sand with the same mix proportion.

## 1. Introduction

Steel slag is mainly composed of impurities in steelmaking charge, slagging materials (limestone, iron ore, dolomite, etc.), eroded furnace linings and oxides of many elements formed by oxidation in the furnace charge [1,2]. The output of steel slag accounts for about 15–20% of the total steel output [3]. Mass production and accumulation of steel slag not only occupy a large area of valuable land resources but also cause some pollution and damage to the environment [4]. The main mineral phase of steel slag is similar to cement, so it has certain potential hydraulic properties and is a potential resource [2,5]. However, the utilization rate of steel slag in China is relatively small at present, not more than 40% [6]. The large-scale and high value-added application of steel slag is limited by the inherent factors of the properties of steel slag: first of all, the composition fluctuation of steel slag brings some difficulties to the stability control when it is used as a raw material for production [7]; secondly, when steel slag is used as a raw material for building materials, because of its high volume expansion, grinding energy consumption and the hidden danger of poor stability, it cannot wholly replace cement [4,8,9]. Improving the utilization rate of steel slag is an effective way to alleviate the load of steel slag on the land and environment, turn waste into treasure and produce certain economic and social benefits.

Steel slag has many applications, such as producing fertilizers, improving soil, treating sewage or as a sinter flux [7,10,11], and it is mainly used as an aggregate or as a substitute for cement in the construction field. Steel slag has certain cementitious activity and is often used as a mineral admixture to replace part of cement. S. Kourounis et al. [12] studied the effect of steel slag replacing part of cement on the properties and hydration of cementitious materials, and it was concluded that steel slag composite cement can reach the strength classes 42.5 and 32.5 of EN 197-1 and steel slag cement had excellent physical properties. Shekhar Saxena et al. [13] used steel slag aggregate to replace the traditional basalt aggregate to prepare concrete, which had a denser microstructure and stronger durability. The compressive strength and flexural strength increased by 33% and 9.8%, respectively, and the elastic modulus increased by 22%. Hisham Qasrawi et al. [14] used steel slag with high stability to replace part of ordinary river sand as an aggregate to prepare concrete. When the steel slag replaced was 15–30%, the 28d compressive strength of concrete increased by 1.1–1.3 times, and the tensile strength was increased by 1.4–2.4 times when the substitution amount was 30–50%. However, the concrete performance would be greatly reduced when the steel slag aggregate was too large. Nicola Faraone et al. [15] found that the larger the particle size of steel slag sand within a certain range, the greater the compressive strength of the test block, and increasing the particle size of steel slag sand could improve the mechanical properties of the mortar test block prepared by steel slag sand. The use of steel slag as an aggregate in pavement could improve its bending resistance, wear resistance and durability, and improve the overall performance of the pavement. The research on steel slag is mainly focused on making a cementitious material as an admixture or fine aggregate, but research on using steel slag as a cementitious material and fine aggregate in concrete is rarely involved.

Using steel slag as a cementing material and fine aggregate in concrete is feasible. Steel slag powder contains f-CaO, active RO phase and other components, which will produce volume expansion in the process of a hydration reaction [9,16,17,18], thus compensating the shrinkage of concrete and reducing or even replacing the use of an expansion agent in concrete [19]. Besides, steel slag sand has weak activity, and the interfacial reaction may occur in the alkaline environment, which may improve the interfacial connection properties. Moreover, compared with river sand, steel slag sand is rough and porous and can produce a “pin effect” [20], further improving the bond strength with cementing materials.

Based on the large amount of steel slag heaped and discarded, and the practical problems of the large-scale and high value-added application of steel slag, it is of great significance to study using steel slag as a cementitious material and fine aggregate. In this paper, the hydration process of alkali-activated materials prepared by steel slag as a cementitious material and fine aggregate was investigated. The phase composition and micro-morphology of hydration products were analyzed by XRD, NMR and SEM, and then the hydration development process was discussed, revealing the corresponding relationship between microstructure and macroscopic properties. It would provide a theoretical basis for the large-scale and high value-added utilization of steel slag.

## 2. Results and Discussion

### 2.1. Hydration Process

#### 2.1.1. Analysis of Hydration Heat

**Figure 1:**
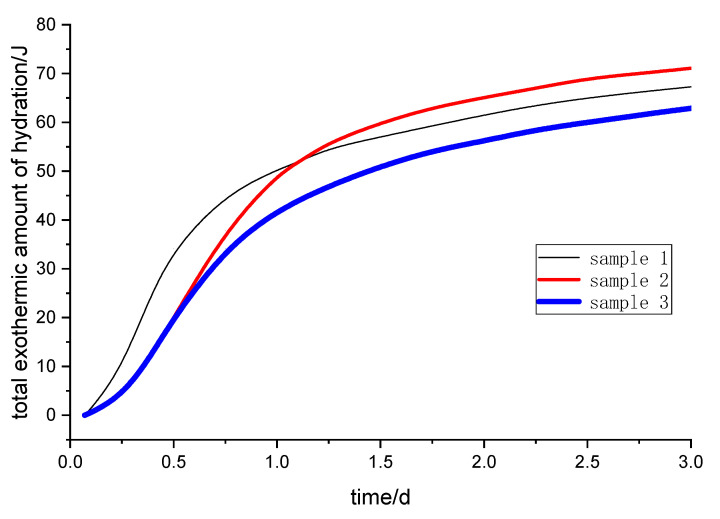
Hydration exothermic curve of different samples.

**Figure 2:**
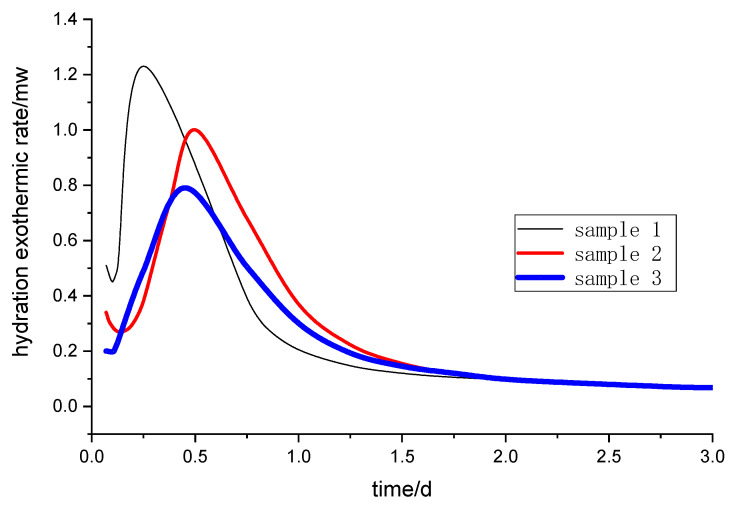
Hydration exothermic rate curve of different samples.

The hydration process of cement concrete is an exothermic chemical reaction. The heat release rate and total heat release energy of the cement hydration process reflect the chemical reaction activity of cementitious materials [21]. The exothermic hydration curve of the alkali-activated steel slag and slag powder composite cementitious material was drawn. The total exothermic amount of hydration and the hydration exothermic rate curve of different samples are shown in Figure 1 and Figure 2, respectively.

In the early stage of curing (from 2 to 30 h), the total heat released is ranked as sample 1, sample 2 and sample 3 (Figure 1). However, after one day of curing, sample 2 has the maximum total heat release. A large amount of heat is released at sample 1 without a retarder, especially within 3 to 12 h. A large number of hydration products such as C-S-H gel are formed quickly, which slows down the diffusion and migration rate of ions in the solution. Some of the powder particles are wrapped in hydration products without even reacting. Compared with sample 1, there are more active substances in sample 2, so the total amount of the exothermic reaction in sample 2 is more than sample 1 after two days.

After a short induction period, the heat release rate of samples rises rapidly and reaches the maximum rate, then the hydration heat release rate gradually slows down and enters a gentle stage after two days (Figure 2). The hydration reaction rates of samples are in the order of sample 1, sample 3 and sample 2, and the maximum reaction rate peaks are reached at 6, 12 and 14 h, respectively. From Figure 1 and Figure 2, the total exothermic amount and the hydration exothermic rate of sample 2 are the largest after one day, indicating that the retarder has a certain retarding effect and hydration-promoting effect on the alkali-activated steel slag and blast furnace slag powder.

#### 2.1.2. Chemically Bound Water Analyzes

Part of the water is involved in the formation of compounds, such as C-S-H gels, during the curing process. However, most of the water is a lubricant between the powders, flowing freely through the concrete pores. In general, free water will evaporate from the material at about 105 °C with 30 min. However, chemically bound water needs a higher temperature because of the bond between the reliable chemically bound water and the substance. After the hydration of silicate materials, most of the hydration products have a large amount of bound water, and the more hydration products there are, the greater the chemically bound water is [12]. Therefore, the chemically bound water is positively correlated with the hydration rate and hydration degree of cementitious material to a certain extent [22].

The content of chemically bound water of the alkali activation reaction process of steel slag-blast furnace slag composites was calculated using Equation (1).

(1) $$W=\left(M_{1}-M_{2}\right)/M_{1}-W_{3}/\left(1-W_{3}\right)$$

**Figure 3:**
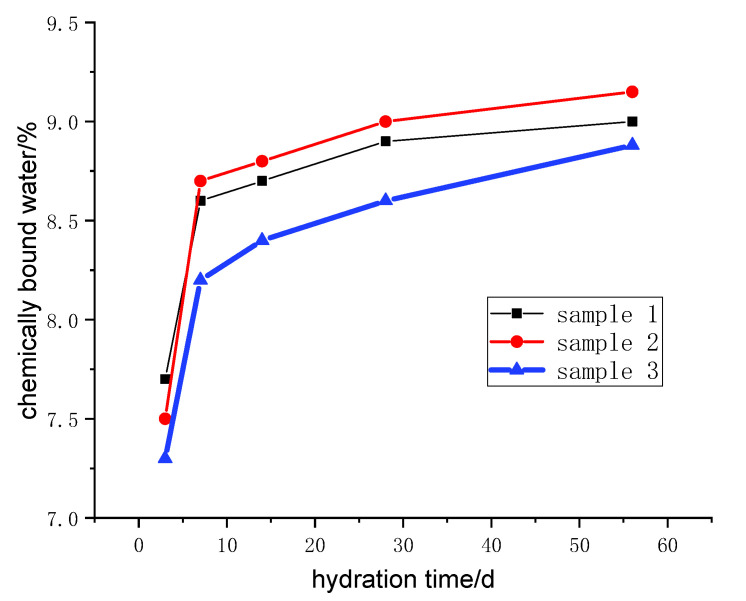
Chemically bound water of samples with different curing ages.

M_1_ and M_2_ are the mass of each hydration sample before and after burning in the muffle furnace. W_3_ is the burning loss of 1g powder mixed in proportion to the ratio under the same conditions. The samples were dried at 105 °C until the weight no longer changed, and then heated from 120 to 600 °C at a heating rate of 10 °C/min. The samples were kept warm for 2 h. The calculated results of the chemically bound water of samples with different curing ages are shown in Figure 3.

With the sample curing process, the chemically bound water increases rapidly in the early curing age (three to seven days), which can be equal to 80 wt.% of the chemically bound water of samples cured for 56 days. Further, the chemically bound water content of sample 2 is the highest in the middle and late stage. Under early curing, steel slag and blast furnace slag powder disintegrate rapidly and form aluminum silicate acid radicals, calcium and other substances [23]. The previously mentioned substances dissolve in the slurry solution, converge and form a large number of C-S-H, C-A-S-H, hydrated calcium aluminate. Under early curing, the concentration of reactants decreases relatively, and the hydration products hinder the diffusion and migration of reaction ions, so the reaction rate decreases greatly, and the growth rate of hydration products slows down.

### 2.2. Phase Analysis of Hydration Products

#### 2.2.1. XRD Analysis

**Figure 4:**
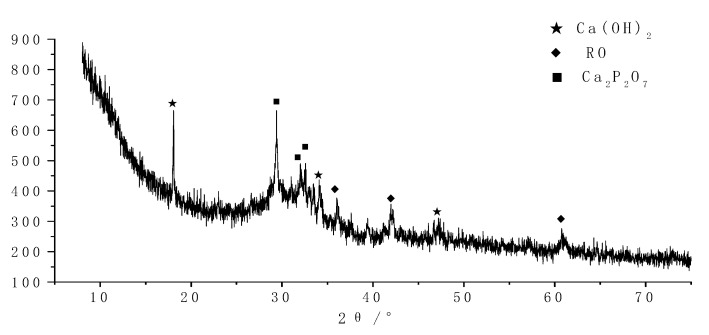
XRD diagram of 3 days of curing samples.

**Figure 5:**
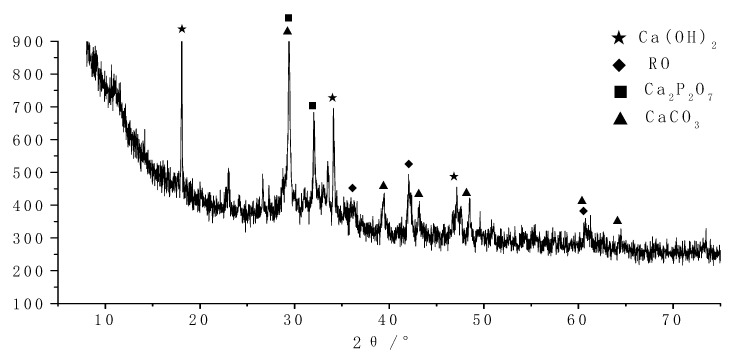
XRD diagram of 28 days of curing samples.

The crystalline phase of all hydrated products was systematically analyzed by the XRD test technique. The results are shown in Figure 4 and Figure 5.

As shown in Figure 4, the crystalline phases are mainly Ca (OH)_2_ and Ca_2_P_2_O_7_ in the early curing products of concrete. Besides, there are a few RO phases (mainly FeO). Meanwhile, a large number of amorphous phases (C-A-S-H gel, etc.) have been observed. After steel slag and blast furnace slag are activated, the powder particles disintegrate rapidly, and the generating ions and ionic groups are dissolved in solution and recombined rapidly. FeO in steel slag can be retained by forming an RO phase with other substances.

As shown in Figure 5, the crystalline is mainly Ca(OH)_2_ and Ca_2_P_2_O_7_ in the later stage of curing products. Besides, a small amount of CaCO_3_ crystal is formed, indicating that the sample is carbonized. Meanwhile, a large number of amorphous phases (C-A-S-H gel, etc.) have also been observed. Simultaneously, the amount of Ca(OH)_2_ increases rather than decreases, because the steel slag activity is weak, and the reaction still occurred in the later hydration stage.

#### 2.2.2. ^27^Al NMR Test and Analysis

**Figure 6:**
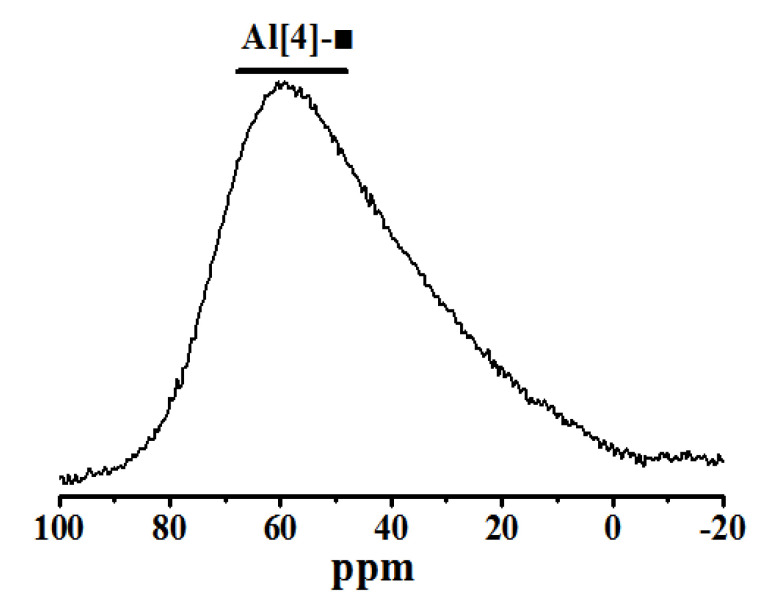
^27^Al NMR spectrum of raw material.

**Figure 7:**
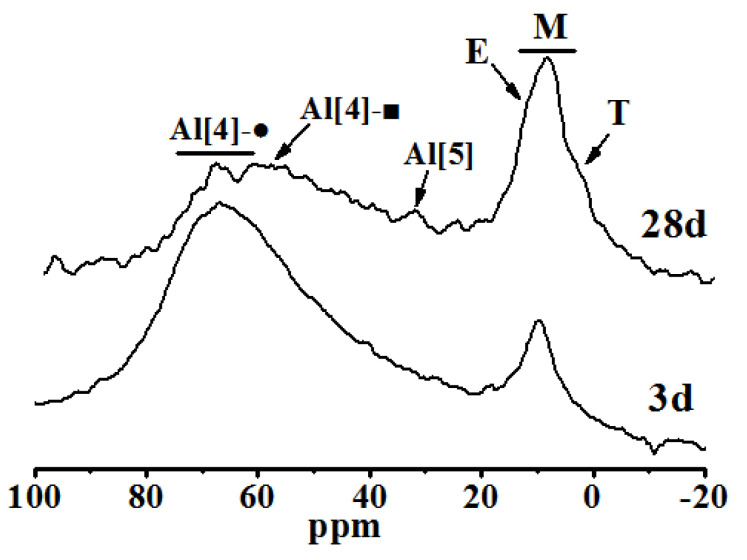
^27^Al NMR spectra of hydrated pastes at different ages.

^27^Al NMR spectra were used to analyze the raw materials and products of steel slag-blast furnace slag before and after curing. The results are shown in Figure 6 and Figure 7.

**Figure 8:**
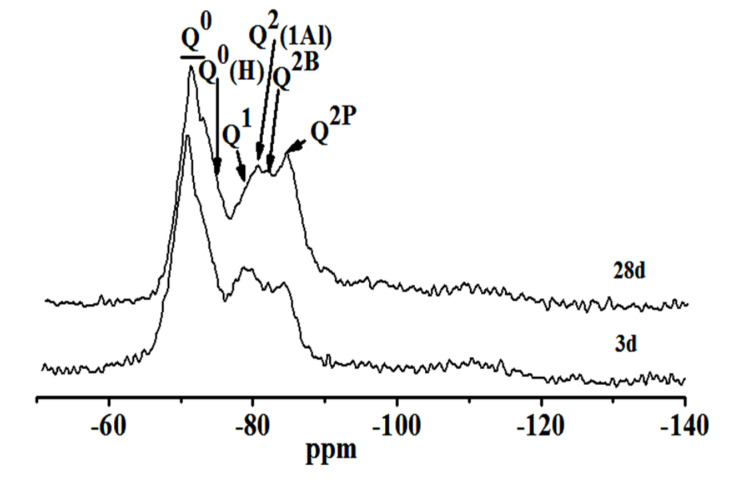
^29^Si NMR spectra of hydrated pastes at different ages.

As shown in Figure 6, the broad asymmetric peak near 59 ppm is related to [AlO4] in the raw material (represented as Al[4]-■), because of the spectral peaks in the raw material overlap. There are three coordination structures of element Al in gel products, four-coordinated aluminum (Al[4]), five-coordinated aluminum (Al[5]) and six-coordinated aluminum (Al[6]) [24,25], as shown in Figure 8. Compared with [SiO_4_], the Al-O bond in [AlO_4_] is 0.1Å longer than the Si-O bond in [SiO_4_]. When Al^3+^ replaces Si^4+^, the tetrahedral oxygen in [AlO_4_] cannot coordinate with the interlayer ions to neutralize the charge, so [AlO_4_] is mainly part of the molecular chain of C-S-H gel [26]. Al[5] mainly enters the interlayer position of the C-S-H structure [27]. Al[6] is primarily adsorbed on the surface and boundary of C-S-H gel particles as a gelatinous aluminate phase or disordered hydrated calcium aluminate and named the third hydrated aluminate (TAH) [28].

In Figure 7, the spectral peak of the chemical shift at 66 ppm belongs to (represented as Al[4]-●) at the bridged position in C-A-S-H, and the peak of the chemical shift at 35.4 ppm corresponds to Al[5] between layers of the C-S-H structure. The chemical shift peaks at 13.1, 9.8 and 5 ppm correspond to AFt (written as E), AFm (represented as M) and TAH (represented as T) [29], respectively. The deconvolution calculation results of Figure 7 are shown in Table 1.

As shown in Figure 6 and Figure 7 and Table 1, some raw material is not fully reacted at both 3 and 28 days of curing. The hydration products that contain Al are mainly C-A-S-H and AFm and a small amount of AFt and TAH, as the same with the XRD analysis. Al^3+^ enters the interlayer and replaces Ca^2+^ in the interlayer position of C-A-S-H to form a small amount of Al[5]. There were still some raw materials that had not reacted completely at 28 days of curing. The contents of AFt, AFm, TAH and interlayer Al[5] of C-A-S-H all increase, but Al[4] decreases. Considering that the activator used in this experiment was Na2SO4, Al may be removed from C-A-S-H to form AFm and Aft in the later hydration stage, which reduces the Al[4] in the C-A-S-H structure [29].

#### 2.2.3. ^29^Si NMR Test and Analysis

The degree of polymerization and substitution of Si was studied by the fitting data of ^29^Si NMR. Figure 8 shows the test results of different curing stages samples. In Figure 8, the chemical degree shift at −68.8–74.5 ppm belongs to the Q^0^ position of unreacted C_3_S and C_2_S, and the chemical shifts at −75.1, −78.7, −80.1, −82.2, −84.5 and −110.5 ppm correspond to Q^0^(H), Q^1^, Q^2^(1Al), Q^2^B and Q^2^P, respectively [26,29]. Q^0^(H) represents unhydrated silicon oxide tetrahedral monomer. Q^2^B and Q^2^P represent the silicon-oxygen tetrahedron of bridging and pairing positions in (C-A-H) C-A-S-H structures, respectively.

Table 2 shows the relative intensity of Q^n^ calculated by deconvolution of the ^29^Si NMR atlas. For the average molecular chain length (MCL), Al[4]/Si were calculated according to Equations (2) and (3) [30]. The results are shown in Table 3.

(2) $$\mathrm{MLC}=2\left[I\left(Q^{1}\right)+I\left(Q^{2}\right)+I\left(Q^{3}\right)+1.5I\left(Q^{2}\left(1\mathrm{Al}\right)+Q^{3}\left(1\mathrm{Al}\right)\right)\right]/I\left(Q^{1}\right)$$

(3) $$\mathrm{Al}\left[4\right]/S_{i}=0.5I\left(Q^{2}\left(1\mathrm{Al}\right)\right)/\left[I\left(Q^{1}\right)+I\left(Q^{2}\right)+Q^{2}\left(1\mathrm{Al}\right)\right)]$$

As curing progresses, the C_2_S, C_3_S and other minerals in steel slag continue to hydrate. The Q^0^ content in raw materials diminishes from 38.5% to 33.1%. Q^0^(H), Q^1^, Q^2^B and Q^2^P increase accordingly. The average molecular chain length of C-S-H(C-A-S-H) and the degree of polymerization increase. However, the content of Q^2^(1Al) decreases, and the ratio of Al[4]/Si diminishes from 0.091 to 0.074, indicating that the degree of Si in C-S-H replaced by Al^3+^ reduces [31]. This is similar to the test result of the ^27^Al NMR spectra.

### 2.3. Microstructure of Hydration Products and Mortar Interface

#### 2.3.1. Microstructure of Hydration Products

The hydration process and mechanism of steel slag-granulated blast furnace slag composite cementitious material were analyzed by SEM-EDS.

(1) 1 day of curing

**Figure 9:**
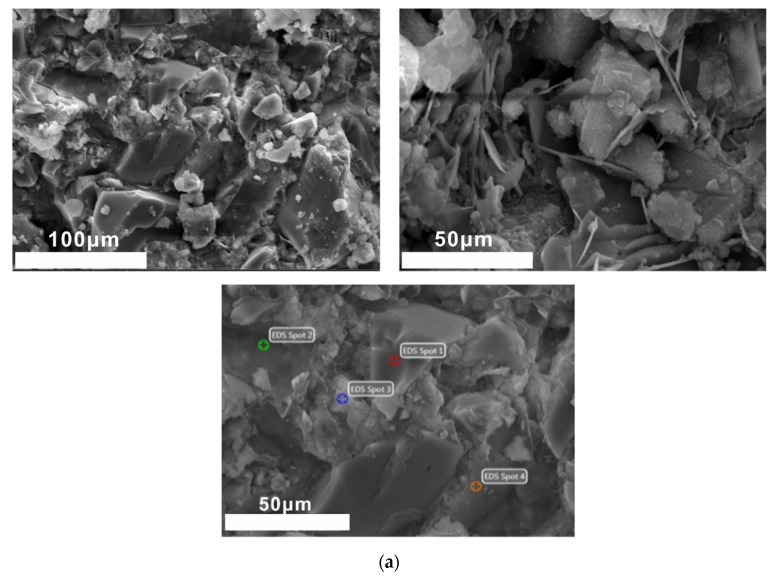

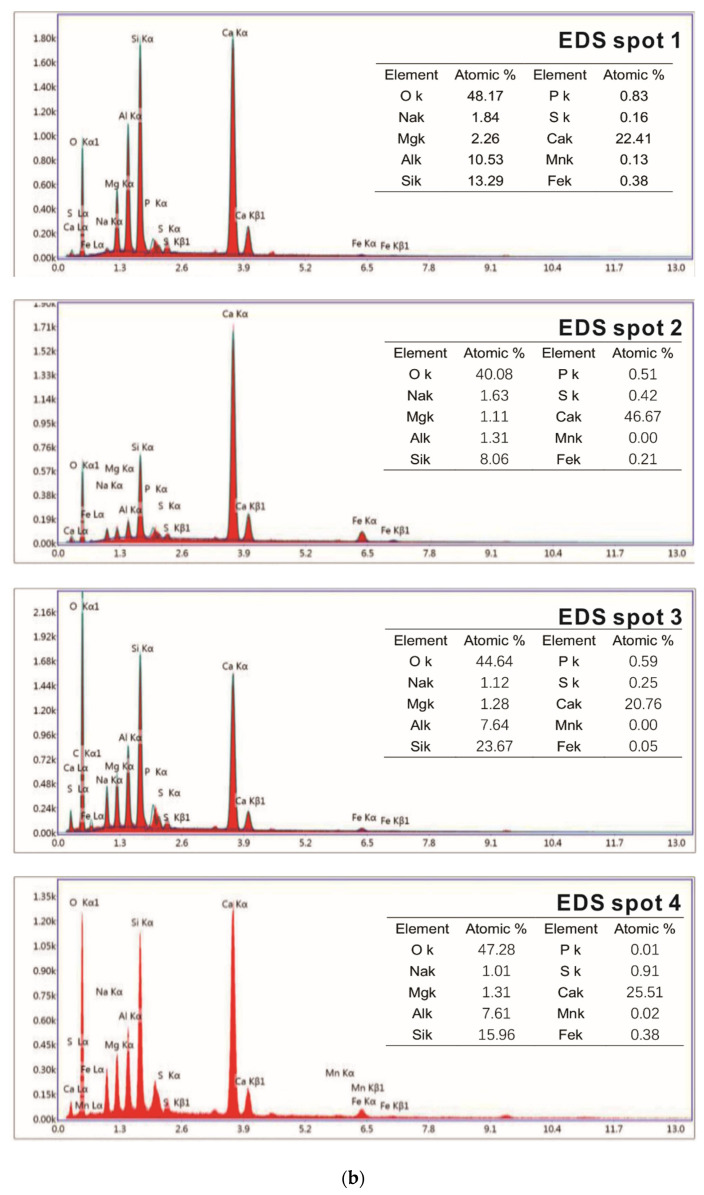
SEM-EDS images of 1 day of curing samples. (**a**) SEM images of 1 day of curing samples, (**b**) EDS images of local region.

It can be seen from Figure 9 that a mass of flocculent C-A-S-H and hexagonal flake Ca(OH)_2_ are formed after one day of curing. Blocky calcium magnesium aluminum feldspar and polygonal tricalcium silicate are observed (the composition is shown in the EDS spectrum of points 1 and 2, respectively). There are evident traces of reactive dissolution in point 3. Combined with its EDS energy spectrum, point 3 is mainly composed of newly formed C-S-H gel and unhydrated aluminosilicate minerals; point 4 is flocculent C-S-H (C-A-S-H), and the C/S is about 1.61.

(2) 3 days of curing

**Figure 10:**
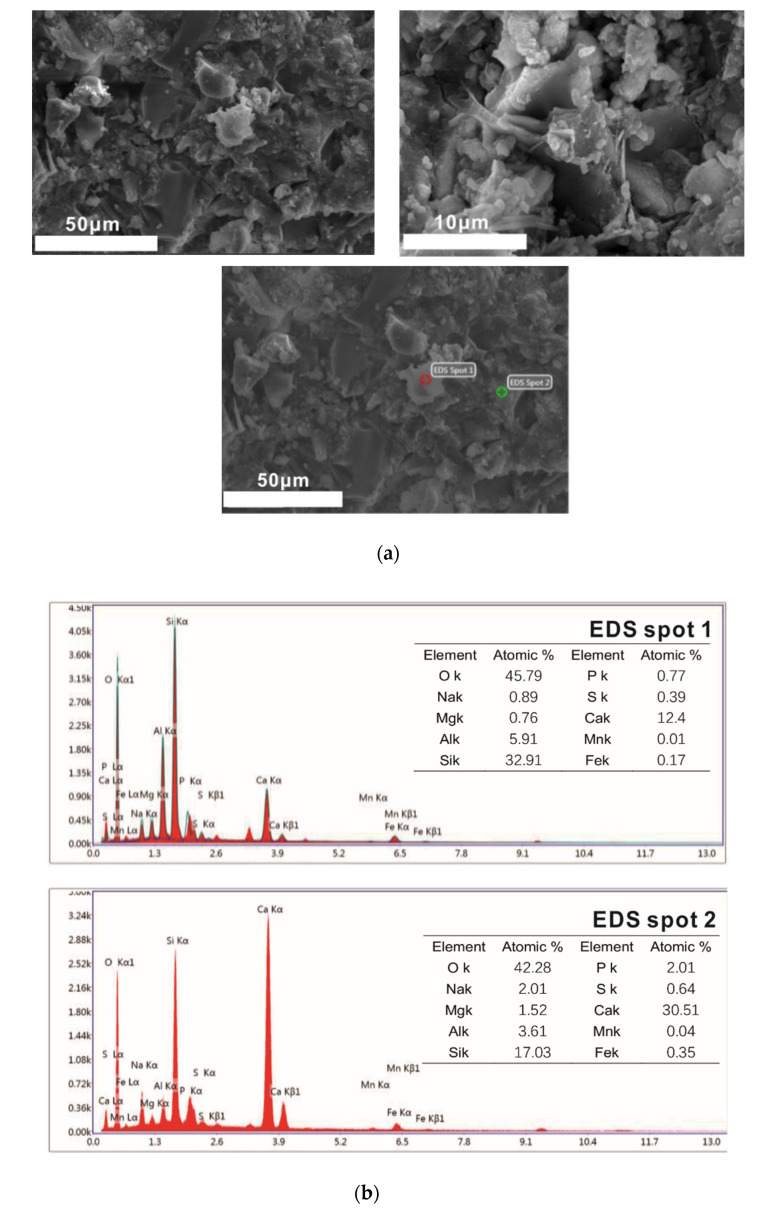
SEM-EDS images of 3 days of curing samples. (**a**) SEM images of 3 days of curing samples. (**b**) EDS images of local region.

As shown in Figure 10, the structures of samples hydrated for three days are dense. The blocky calcium magnesium alumina feldspar is tightly surrounded by hydration products. The loose accumulated flake Ca(OH)_2_ has grown into a thick and dense block and is closely bound to the C-S-H (C-A-S-H) gel. Acicular ettringite grows in a small number of voids. As shown in the EDS energy spectrum and morphology of point 1, there are still reactive residues in samples after curing for three days. Point 2 is the hydration product in the C-S-H gel, and its C/S is about 1.78 according to its EDS energy spectrum data.

(3) 28 day of curing

**Figure 11:**
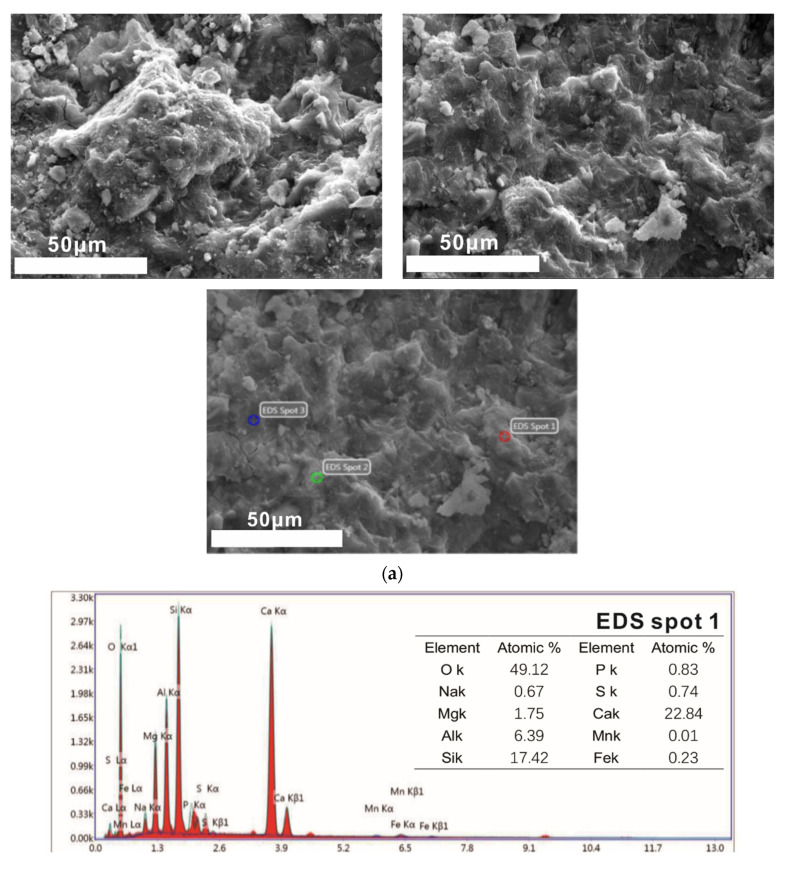

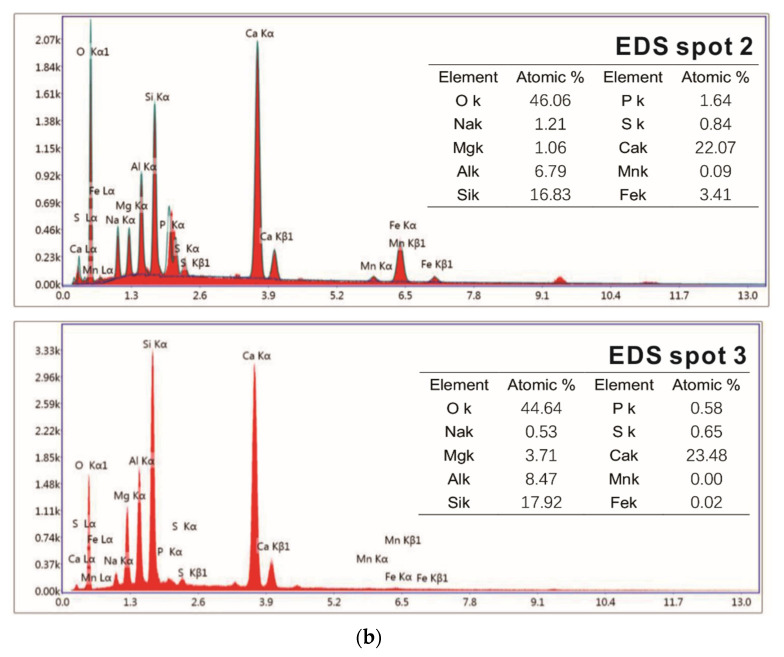
SEM-EDS of 28 days of curing samples. (**a**) SEM images of 28 days of curing samples. (**b**) EDS images of local region.

It can be seen from Figure 11a that the 28 days of curing samples have been connected into a whole by the C-S-H gel. There are some white particles of unequal size and irregular shape on the surface. The existence of blocky calcium magnesium alumina feldspar, needle rod ettringite and flake or plate Ca(OH)_2_ cannot be found, and the compactness of the sample has been further improved.

As shown in Figure 11b, the major elements of hydration products are calcium, silicon, aluminum, magnesium, sodium, oxygen and other elements. At point 2, there are not only natural hydration products in the C-S-H (C-A-S-H) gel, but also particular hydration products such as Mg_x_ (Mg, Fe)_3_(Si, Al)_4_O_10_(OH)_2_·4H_2_O. The main substance in the area of point 1 and point 3 is C-S-H (C-A-S-H) gel, but there are also some substances containing magnesium. The C/S of the C-S-H gel of the hydration sample for 28 days is about 1.31.

From the above SEM-EDS test and analysis, it can be found that with the extension of curing, the amount of C-S-H (C-A-S-H) gel increases, and finally other substances are wrapped up and connected into a dense whole. C/S increases at first and then decreases, which may be due to the high content of calcium oxide in steel slag powder, and its hydration activity is lower than that of granulated blast furnace slag powder [32].

#### 2.3.2. Microstructure of Interfacial Transition Zone

**Figure 12:**
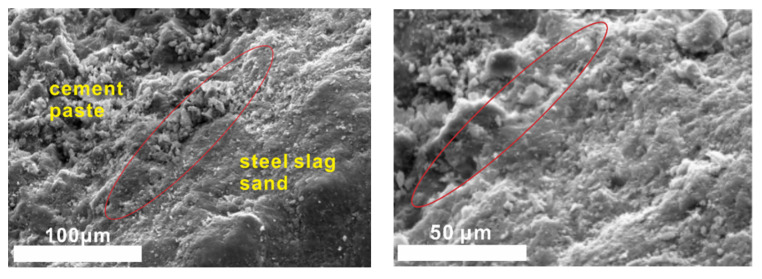
SEM images of samples hydrated for 28 days with steel slag sand as the aggregate.

**Figure 13:**
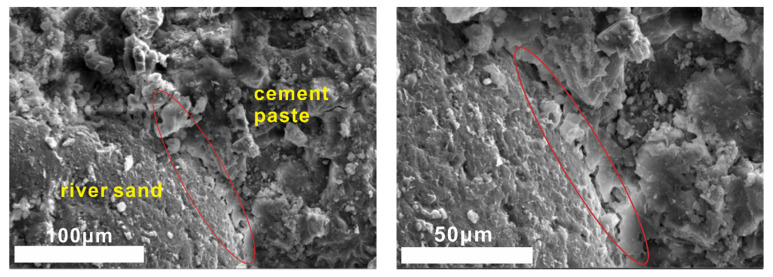
SEM images of samples hydrated for 28 days with river sand as the aggregate.

Steel slag sand is mainly composed of oxides such as SiO_2_, CaO and Al_2_O_3_ and has weak hydration activity compared with ordinary river sand (consisting mainly of SiO_2_). The mortar samples were taken out at the age of 28 days to observe the interface morphology of cement stone, steel slag sand and river sand by scanning electron microscope (Figure 12 and Figure 13).

**Figure 14:**
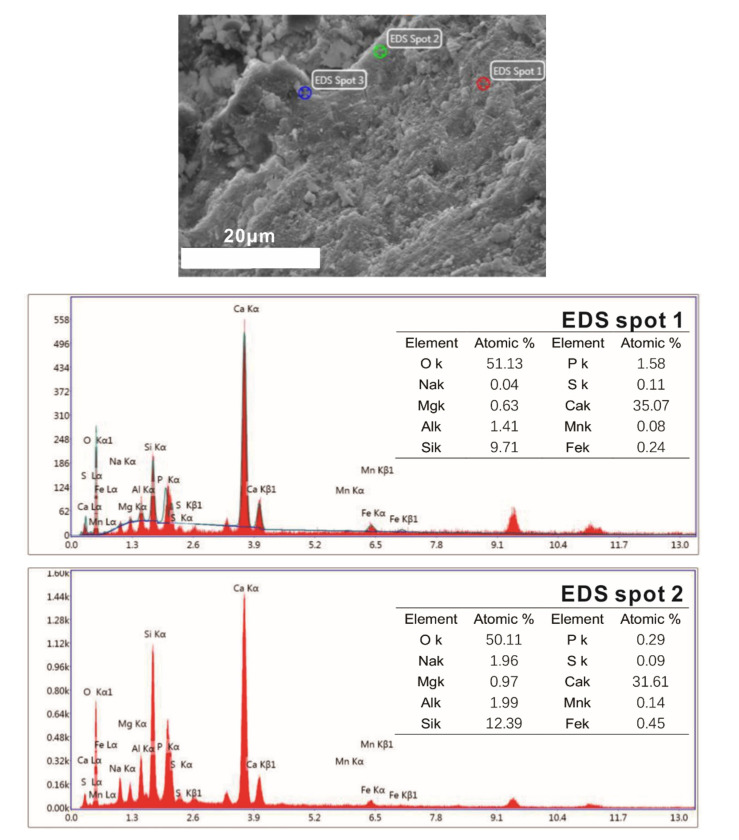

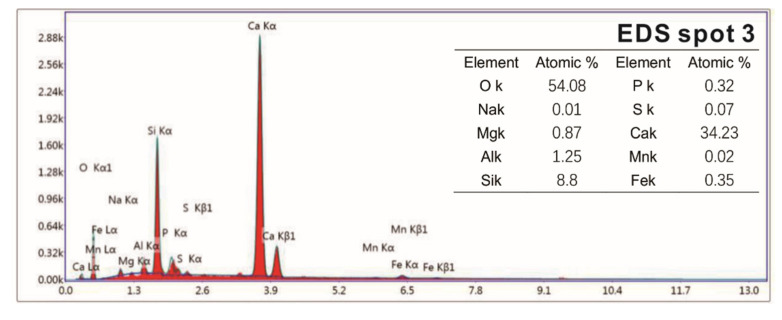
Surface morphology and energy spectra of steel slag sand.

There is no obvious interface between mortar cement paste and steel slag sand when steel slag sand is used as a fine aggregate in samples cured for 28 days from Figure 12 and Figure 13. Steel slag sand is closely connected with cement paste, while river sand and the mortar cement stone with river sand as the fine aggregate have an obvious interface. The interface results in internal defects (pores and microcracks), which will adversely affect the strength of the corresponding samples. At the same time, a small amount of hydration products form on the surface of steel slag sand, while the surface of river sand is smooth and almost no other substances exist. From the results of the energy spectrum analysis in Figure 14, the Na/Mg is close to 2, while the Na/Mg of steel slag sand is about 0, indicating that a small amount of hydration products containing sodium are formed.

From Figure 12 and Figure 14, the interface between cement paste and steel slag sand is compact, and there is no weak area formed by the enrichment of Ca(OH)_2_, which is different from the interface transition zone of ordinary cement concrete aggregate [33]. The interface is composed of C-S-H (C-A-S-H) gel and Ca(OH)_2_ crystal through the energy spectrum analysis. With Ca(OH)_2_ crystal as the framework, C-S-H gel is wrapped around it, and finally a dense cementitious structure is formed [34]. Following other conditions, the 28-day compressive strength of steel slag sand mortar is 49.2 MPa, while that of river sand mortar is 47.3 Mpa. This is due to the rough surface of steel slag sand and the closer connection with cement paste, and the partial hydration reaction on the surface of steel slag sand, which is connected with cement paste as a whole.

## 3. Experimental Materials and Test

### 3.1. Raw Material

Steel slag (basic oxygen steel slag) was produced by Wuhan Iron and Steel Group Co., Ltd. (Wuhan, China), the steel slag was broken, then the large particles were used as steel slag sand, and the small particles were made into steel slag powder (SS) by drying and grinding. Granulated blast furnace slag powder (BFS) was created by WISCO Green Metallurgical Slag Co., Ltd. (Wuhan, China), and the expansion agent (EA) came from Tianjin Baoming Company (Tianjin, China). The chemical composition of all raw powder is shown in Table 4. The screening of steel slag sand and river sand is shown in Table 5. The alkali activator is composed of 1 wt.% NaOH, 1 wt.% Na_2_SO_4_ and 4 wt.% sodium silicate.

### 3.2. Sample Preparation

The water/binder ratio is controlled to 0.3 by adding 0.3 wt.% water reducing agent during the preparation of paste samples, and the total amount of cementitious material is 500 kg/m^3^. The mix proportion of cementitious materials is shown in Table 6.

According to the mix proportion in Table 3, the powder raw materials were mixed evenly, then the liquid raw materials (activator, water reducer, retarder and water) were added, fully stirred to form the slurry, and then cast to prepare the paste sample. The aggregate river sand (1500 kg/m^3^) or steel slag sand (1500 kg/m^3^) was added into the slurry prepared by the mix proportion of sample 2, mixed evenly and then cast to form mortar samples. Both the paste samples and mortar samples were molded with a 40 × 40 × 40 cm mold, and then demolded after 24 h. All samples were cured at 25 ± 2 °C and 95% humidity for different times. Mortar samples were prepared according to <GB/T 25181-2010> [35].

### 3.3. Test and Characterization

The exothermic processes of the steel slag-granulated blast furnace slag powder system were measured by a hydration microcalorimeter (TAM Air, TA Instruments, New Castle, NY, USA) at 20 °C. The steel slag powder was mixed with other raw materials to prepare a paste, then the resulting paste was injected into an ampoule that was then put into a calorimeter. The heat flow record time is 3 days.

Samples of different reaction ages were broken into pieces and placed in alcohol to stop hydration. Then, the phases’ composition of hydration products was investigated by an X-ray diffraction (XRD) diagram (D8 ADVANCE, Bruker, Karlsruhe, German), with continuous scanning, the 2θ range of 5° to 75°, a scanning rate of 5°/min and a step width of 0.0195°. The internal structure of the hydration products of the system that reacted for 3 days and 28 days was tested by a nuclear magnetic resonance (NMR) instrument (AVANGE III HD400MHz, Bruker, Karlsruhe, German). The morphology structure and micro-elements of hydration products were observed by scanning electron microscope (SEM) patterns (ULTRAPLUS-43-13, Carl Zeiss, Jena, German) and accompanying energy disperse spectroscopy (EDS) patterns (X-max 50, Oxford Instruments, Oxfordshire, UK).

The content of the test and characterization in this study is shown in Table 7.

## 4. Conclusions

In this paper, the hydration process of alkali-activated materials prepared by steel slag as a cementing material and fine aggregate was studied. The phase composition and micro-morphology of the hydration products were analyzed. The development process of hydration and the corresponding relationship between microstructure and macro-properties were discussed. Based on the above analysis and discussion, the following conclusions are drawn:

(1) The retarder has a certain retarding effect on the alkali-activated steel slag and blast furnace slag powder cementitious system, and the main hydration products are crystalline Ca(OH)_2_ and amorphous-phase C-A-S-H gel.

(2) With the extension of the curing age, the amount of C-S-H (C-A-S-H) gel and the average molecular chain length increase, so the degree of polymerization increases. Al[4]/Si decreases, so the degree of Si in C-S-H replaced by Al^3+^ reduces, while C/S increases first and then decreases with the progress of hydration. Further, the structure of cement paste is compact.

(3) The interface between steel slag sand and cement paste is denser than that of river sand, since the surface of steel slag sand is partially hydrated to form C-A-S-H gel and Ca(OH)_2_. As a result, the compressive strength of concrete prepared by steel slag sand is higher than that of river sand at the same mix ratio.

## Figures and Tables

**Table 1:** Calculation results of deconvolution of ^27^Al NMR spectra.

Sample	Relative Strength Value/%					
	Al[4]-■	Al[4]-●	Al[5]	E	M	T
3d	45.06	40.01	1.29	2.18	9.73	1.73
28d	26.17	31.44	4.05	11.06	21.04	6.24

**Table 2:** Calculation results of deconvolution of Q^n^ in ^29^Si NMR spectra.

Q^n^Sample	Relative Strength Value/%					
	Q^0^	Q^0^(H)	Q^1^	Q^2^(1Al)	Q^2^B	Q^2^P
3 d	38.5	9.4	19.8	9.3	7.3	14.7
28 d	33.1	9.8	20.5	10.2	8.6	17.8

**Table 3:** Molecular chain length (MCL) and Al[4]/Si at different ages.

ParameterAge	MCL	Al[4]/Si
3 d	5.90	0.105
28 d	6.07	0.089

**Table 4:** Chemical composition of powder (wt.%).

	SiO_2_	Fe_2_O_3_	Al_2_O_3_	MgO	K_2_O	Na_2_O	CaO	SO_3_	P_2_O_5_	LOI
SS	20.66	15.72	6.41	4.58	0.16	0.11	41.86	1.37	1.31	2.65
BFS	38.50	0.30	11.90	7.30	0.60	0.50	40.28	0.28	0.16	0.30
EA	13.46	1.80	5.84	4.14	0.41	0.12	63.75	—	—	5.10

Note: the ‘—’ represents that the corresponding component has not been detected.

**Table 5:** Screening of steel slag sand and river sand.

Particle Size/mm	Fractional Sieve Residual Percentage/%		Cumulative Sieve Residual Percentage/%	
	Steel Slag Sand	River Sand	Steel Slag Sand	River Sand
4.75	—	—	—	—
2.36	14.7	3.7	14.7.	3.7
1.18	26.8	12.7	41.5	16.4
0.6	25.8	48.5	67.3	64.9
0.3	24.7	34.1	92.0	99.0
0.15	5.2	0.6	97.2	99.6
Sieve bottom	2.4	0.1	99.6	99.7

Note: the ‘—’ represents that the percentage of particle size between 2.36 and 4.75 is 0.

**Table 6:** The mix proportion of cementitious materials (kg/m^3^).

Sample	SS	BFS	EA	Activator/wt.%	Water reducer/wt.%	Retarder/wt.‰	Water
1	189.1	283.6	27.3	1 NaOH, 1 Na_2_SO_4_, 4 sodium silicate	0.3	—	140
2	189.1	283.6	27.3	1 NaOH, 1 Na_2_SO_4_, 4 sodium silicate	0.3	1	140
3	189.1	283.6	—	1 NaOH, 1 Na_2_SO_4_, 4 sodium silicate	0.3	1	140

Note: the ‘—’ represents that the mix proportion of the corresponding raw materials is 0.

**Table 7:** Content of the test and characterization.

Test	Sample	Test Analysis Purposes
Hydration Heat	Paste	Hydration process
XRD	Paste	Phase composition
NMR	Paste, raw materials	Internal structure
SEM-EDS	Paste, mortar	Morphology structure
